# Information Thermodynamics of the Cell Signal Transduction as a Szilard Engine

**DOI:** 10.3390/e20040224

**Published:** 2018-03-26

**Authors:** Tatsuaki Tsuruyama

**Affiliations:** Clinical Research Center for Medical Equipment Development, Department of Pathology, Kyoto University Hospital, Shogoin-Kawahara-cho 54, Sakyo-ku, Kyoto 606-8057, Japan; tsuruyam@kuhp.kyoto-u.ac.jp; Tel./Fax: +81-75-751-3111

**Keywords:** Szilard engine, mutual entropy, signal transduction, information thermodynamics, fluctuation theorem

## Abstract

A cell signaling system is in a non-equilibrium state, and it includes multistep biochemical signaling cascades (BSCs), which involve phosphorylation of signaling molecules, such as mitogen-activated protein kinase (MAPK) pathways. In this study, the author considered signal transduction description using information thermodynamic theory. The ideal BSCs can be considered one type of the Szilard engine, and the presumed feedback controller, Maxwell’s demon, can extract the work during signal transduction. In this model, the mutual entropy and chemical potential of the signal molecules can be redefined by the extracted chemical work in a *mechanicochemical* model, Szilard engine, of BSC. In conclusion, signal transduction is computable using the information thermodynamic method.

## 1. Introduction

Biological systems exist in an open and stable non-equilibrium state, homeostasis, which fluctuates. These systems most likely utilize fluctuations for various cellular activities, molecular motors [1,2], and possibly for the signal transduction [3,4,5,6], i.e., the transmission of biological information. Biological signal transduction systems are characterized by the presence of biochemical signaling cascades (BSCs). These BSCs are interconnected, forming a network. For the evaluation of environmental stimuli and their consequences, different computational methodologies and the field of systems biology have been developed. 

Signal transduction refers to the sequential biochemical reactions that include the modification or demodification of multi-enzymatic molecules. The enzymes may be the substrates in the subsequent reactions, and the modification sequence may be considered the signal flow sequence with the form change of different modified proteins [7]. The sequential response to the environmental stimuli is mediated by many intracellular metabolites, such as adenosine triphosphate (ATP), and its modified form, cyclic AMP, represents one of the second messengers [8].

The biological systems maintain homeostasis, steady isothermal and isovolumic states. The author considered the feedback systems, a unique property of the biological systems, allowing the maintenance of the steady state in the system. BSC can be considered the utilization of the tentative increase in fluctuation as the mediator of the signal transmission [7]. The feedback contributes to the decrease in the fluctuation. If the feedback system is operated by a feedback controller, termed Maxwell’s demon, a biological signaling system can be simplified and presented as a model to obtain the definite computation of the signal transduction. According to the previously proposed information thermodynamic theory [5,6], the upper limit of the average work <*w*> that can be extracted from the cyclic system by the feedback controller depends on the system temperature *T*, Boltzmann constant *k_B_*, free energy change Δ*F*, and the mutual information *H*, which is measured by the feedback controller, as shown in Inequality (1) [9]:

(1) $$\langle w\rangle \leq \Delta F+k_{B}TH.$$

This inequality represents the generalized second law of thermodynamics [2,10]. In order to achieve equality in Inequality (1), the feedback process is quasi-static, and the usage of the acquired information from the Szilard engine should be non-wasteful [11]. In this study, the author considered the extra-work utilized for the signal transduction in the informational biological thermodynamic system, which fluctuates around a steady state, i.e., homeostasis. For the isothermal and isovolumic biological system, Inequality (1) can be simplified to:

(2) $$\langle w\rangle \leq k_{B}TH.$$

The specific aim of this study was to develop a theoretical formulation of the chemical potential for signal transduction, based on the thermodynamic information theory, to be applied in the investigations of signal transduction.

## 2. A Model Signaling Cascade

If an isothermal and isovolumetric biological system is considered, a model of the BSC, consisting of *j* and the reverse −*j* steps can be constructed (1 ≤ *j* ≤ *n*).

(3) $$\begin{array}{l} X_{1}(R)+L\to X_{1}-L*:1^{st}\\ X_{1}-L*\to X_{1}+L:-1^{st}\\ X_{1}-L+X_{2}+A\to X_{1}-L+X_{2}*+D:2^{nd}\\ X_{2}*+Ph_{2}\to X_{2}+Pi\to -2^{nd}\\ \cdots \\ X_{j}*+X_{j+1}+A\to X_{j}*+X_{j+1}*+D:\to j^{th}\;\\ X_{j+1}*+Ph_{j}\to X_{j+1}+Pi\;:\to -j^{th}\\ \cdots \\ X_{n-1}*+X_{n}+A\to X_{n-1}*+X_{n}*+D:\;\to n-1^{th}\;\\ X_{n}*+Ph_{n}\to X_{n}+D:\leftarrow -{\left(n-1\right)}^{th}\\ X_{n}*+DNA+RNApol+N\;ribonucleotide⇌\\ X_{n}+DNA*+RNApol\;:\;\to n^{th},\leftarrow -n^{th} \end{array}$$

**Figure 1:**
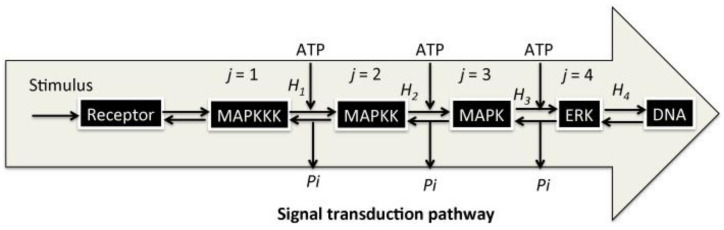
Schematic showing an example of signal transduction pathway. MAPKKK, Mitogen-activated Protein (MAP) kinase kinase kinase; MAPKK, MAP kinase kinase; MAPK, MAP kinase; ERK, extracellular signal-regulated kinase; DNA, deoxyribonucleic acid. The right pointing arrows represent the direction of BSC and the left pointing arrows represent the reverse direction. Adenosine triphosphate (ATP) represents the supplied adenosine triphosphate from the outside world and *Pi* represents the released inorganic phosphate from the pathway to the outside world. Stimulus represents binding of growth factor or other chemokines to the receptor. *H_j_* (*j* = 1, 2, 3, 4) represents the transmitted mutual information along the direction.

Each step represents an enzymatic modification/demodification of the signaling molecules in the cytoplasm, maintained by a chemical reservoir that provides signaling molecules, such as ATP (symbol *A* in the Formula (3)). ATP is a well-known mediator of signal transduction, and it is hydrolyzed into adenosine diphosphate (ADP; *D* in the Formula (3)) and an inorganic phosphate (*Pi*), which is utilized for the modification of the amino acid residue of *X_j_*. Here, *X_j_* and X_j_* denote a signaling molecule, either unmodified (inactive) or modified (active) by the signaling molecule, respectively. The first reaction (*j* = 1) in the BSC represents the uptake or binding of an extracellular molecule, the ligand (L), by *X*_1_, which represents a receptor (R) located on the cellular membrane, which is involved in sensing of the external stimulation by L. Afterward, *X*_1_ − L promotes the modification of *X*_2_ in the cytoplasm into *X*_2_*, phosphorylated by *Pi* from ATP, and ADP is produced. The BSC processes continue in this manner, such that the *j^th^* signaling molecule, X_j_*, induces the modification of *X_j_*_+1_ into *X_j_*_+1_*. Following the final step, the signaling molecule X_n_* translocates to the cell nucleus, where it binds to the promoter region of DNA and induces gene transcription of mRNA from ribonucleotides in the final step in (3). During the BSC steps, demodification of *X_j_*_+1_* into *X_j_*_+1_ occurs through an enzymatic reaction mediated by a phosphatase (*Ph_j_*; 1 ≤ *j* ≤ *n* − 1), in which *Pi* is released. A pre-stimulation steady state is recovered in this manner [7] (Figure 1). 

The occurrence probability (*p_j_*), which represents the selection probability of the *j^th^* step using the signaling molecule concentration, *X_j_* is defined respectively:

(4) $$p_{j}=X_{j}/X$$

with

(5) $$p_{j}*=X_{j}*/X$$

and

(6) $$\sum_{j=1}^{n}\left(p_{j}+p_{j}*\right)=\;1.$$

Here, the author introduces *X* that represents the total concentration of the signaling molecules.

(7) $$X=\sum_{j=1}^{n}X_{j}+X_{j}*=const.$$

Because the sum of the concentrations is considered constant, protein production is relatively slower than the signal transduction step. Therefore,

(8) $$p_{j}+p_{j}*=p_{j}{}^{0}=const.$$

## 3. Signaling Cascade as a Szilard Engine

**Figure 2:**
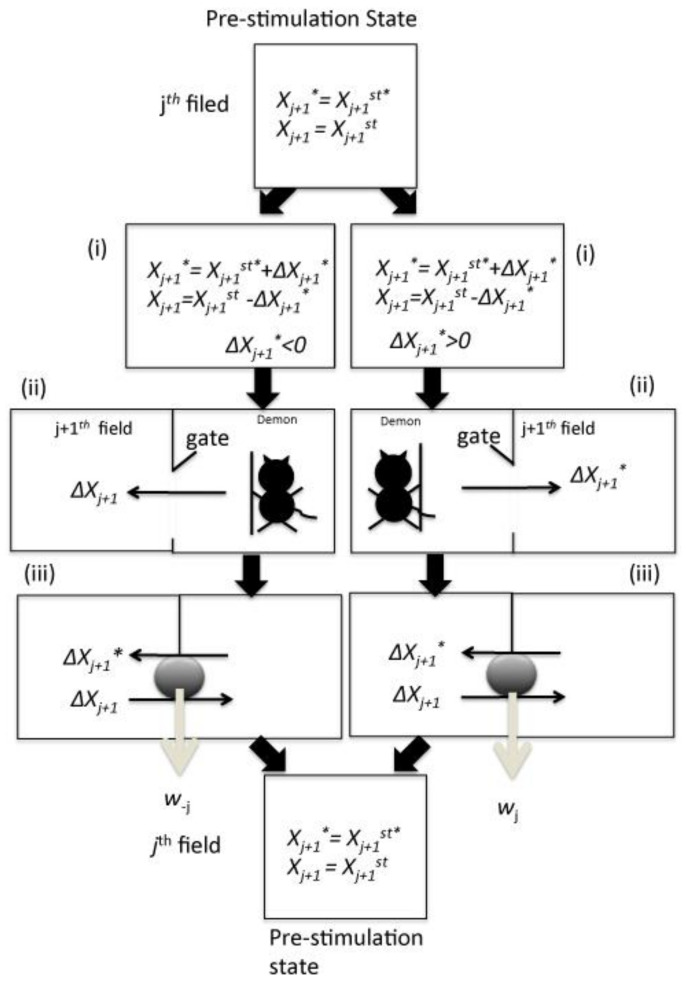
Schematic showing feedback controller processes. (**i**) The feedback controller observes the increase of *X_j_*_+1_*. (**ii**) The controller opens the gate for the increased Δ*X_j_*_+1_* or for the increased Δ*X_j_*_+1_ to enter the (*j* + 1)*^th^* field from the *j^th^* field, to prevent the signal from proceeding further. The *j^th^* field in the system recovers to the initial state in this reaction cycle. (**iii**) The chemical work (*w_j_*) can be extracted by the backflow of Δ*X_j_*_+1_* of *X_j_*_+1_* from (*j* + 1)*^th^* field to the *j^th^* field. If the feedback controller observes the increase of *X_j_*_+1_, the controller opens the back gate and the next steps follow and the chemical work (*w_−j_*) can be extracted by the backflow of Δ*X_j_*_+1_ of *X_j_*_+1_ from the (*j* + 1)*^th^* field to *j^th^* field. The gray globule on the barrier represents the machinery between the steps.

Here, the author hypothesized that the feedback controller determines the activation or inactivation of signaling molecules in contact with an ATP chemical reservoir, with ATP freely transferred between the reservoir and the individual *j^th^* reaction field. The cell signaling system can be divided into an *n* number of fields, corresponding to the individual *j^th^* step (1 ≤ *j* ≤ *n*), for the formation of the BSC from the 1*^st^* to *n^th^* step. In a biochemical system, signaling molecules are macromolecules localized in the cytoplasm, and, since their diffusion rate is sufficiently slow, the signaling reaction is considered a localized system as well. In the current model, each field contains all *X_j_*_+1_* and *X_j_*_+1_ species (1 ≤ *j* ≤ *n −* 1), with the concentrations identical to those of *X_j_*_+1_*^st^ and *X_j_*_+1_*^st^*, respectively, at the initial state, when the stimuli are absent. The lowercase subscript *st* on the right of *X_j_*_+1_*^st^ and *X_j_*_+1_*^st^* represents the steady-state concentration. In this system, the feedback controller has the potential to recognize the ratio of signaling molecule concentration differences between the initial and final states in the *j^th^* field. Subsequently, the controller provides feedback by selecting the transferring of *X_j_*_+1_* or *X_j_*_+1_, to determine the orientation of the signal transduction. Each individual *j^th^* step in Formula (3), consists of a four-step chemical cycle (Figure 2), which can be summarized as follows:

(i) The feedback controller measures the changes in the concentration of the active signaling molecule *X_j_*_+1_* in the *j^th^* field.
(ii) If *X_j_*_+1_* concentration increases from *X_j_*_+1_^st^* to *X_j_*_+1_^st^* + Δ*X_j_*_+1_* (Δ*X_j_*_+1_* > 0), the *j^th^* (1 ≤ *j* ≤ *n*) step proceeds in the same signaling direction, while the feedback controller introduces Δ*X_j_*_+1_* of *X_j_*_+1_* to the (*j* + 1)*^th^* field from the *j^th^* field by opening the forward gate in the *j^th^* field to the (*j* + 1)*^th^* field. In contrast, if *X_j_*_+1_* concentration decreases from *X_j_*_+1_^st^* to *X_j_*_+1_^st^* − Δ*X_j_*_+1_* (Δ*X_j_*_+1_* > 0), the *j^th^* step proceeds in the opposite direction of BSC. In that case, the controller introduces Δ*X_j_* of *X_j_* to the (*j* + 1)*^th^* field from the *j^th^* field by opening the back gate.
(iii) Subsequently, *X_j_*_+1_* flows back with the forward transfer of *X_j_*_+1_ from the (*j* + 1)*^th^* field to the *j^th^* field due to the mixing entropy gradient. This *X_j_*_+1_* can rotate the machinery that can extract chemical work equivalent to *w_j_*. If *X_j_* flows back from the (*j* + 1)*^th^* field to the *j^th^* field simultaneously with the transfer of *X_j_*_+1_* due to the mixing entropy gradient with the rotation of the molecular machinery, this molecule can extract the chemical work equivalent to *w*_−*j*_.
(iv) In the (*j* + 1)*^th^* to the (*j* + 2)*^th^* field, a similar reaction is initiated for signal transduction in an identical or the opposite orientation.

During signaling transduction in a cell, the total concentration of signaling molecules is kept nearly constant (*X_j_*_+1_ + *X_j_*_+1_* = const.), and here, the author can use:

(9) $$dX_{j+1}+\;dX_{j+1}*=\;0.$$

## 4. Mutual Entropy and Chemical Work by a Model of Szilard Engine

We further investigated whether cell signal transduction can be modeled as a Szilard engine [11]. As a well-known example, let us consider a type of Szilard engine in which a single particle is enclosed in a field in contact with a heat bath. To obtain the information *H*, irrespective of its location in the left or right side of the field, a barrier was inserted in the middle of the field. The information *I* can then be recorded by a feedback controller and utilized as a resource for the extraction of work through the isothermal expansion of the left or right side. This can be done by moving the barrier in order to recover the initial state. The movement orientation of the barrier is determined by the obtained information, which represents a feedback process.

Next, the author subsequently considered the amount of information *H_j_* that can be transmitted, using this model system of signal transduction. The cyclic reaction, including the exchange between *X_j_*_+1_ and *X_j_*_+1_*, satisfies the requirements of the Szilard engine, because the acquired data on the *X_j_*_+1_* change in (ii) is used as a resource for the quasi-static work in (iii). In general, the upper limit of the extracted average chemical work by the machinery between the *j^th^* and *j* + 1*^th^* reaction field, <*w_j_>*, can be obtained based on the previous research on Helmholtz free energy Δ*F_j_* and mutual information *H_j_* [2,10,12,13,14,15,16]:

(10) $$\langle w_{j}\rangle \leq -\Delta F_{j}+k_{B}TH_{j}$$

In (10), the lowercase *j* represents the number of the cyclic reaction step in the BSC. Therefore, the extracted average chemical work that can be extracted from a Szilard engine was given by (10):

(11) $$\langle w_{j}\rangle =k_{B}TH_{j}.$$

The mutual entropy that is received by feedback controller is substantially equivalent to the difference mixing entropy between *I_j_* at the *j^th^* reaction field and *I*_*j*+1_ at the *j*+1*^th^* reaction field. Considering the entropy current arising from the difference in the mixing entropy of *j^th^*, to (*j* + 1)*^th^* steps consisting of *X*_*j*+1_* and *X*_*j*+1_ differences, the mixing entropy of activated *j^th^* step is described as follows [7]:

(12) $$I_{j}=-X\left[\left(p_{j+1}+\Delta p_{j+1}\right)\log\left(p_{j+1}+\Delta p_{j+1}\right)+\left(p_{j+1}*+\Delta p_{j+1}*\right)\log\left(p_{j+1}*+\Delta p_{j+1}*\right)\right]$$

where Δ*p*_*j*+1_* and Δ*p*_*j*+1_ denote the fluctuations of the occurrence probability. In the (*j* + 1)*^th^* step, in the absence of *X_j_* and X_j_* fluctuations, the following calculation can be applied:

(13) $$I_{j+1}=-X\left[p_{j+1}\log p_{j+1}+p_{j+1}*\log p_{j+1}*\right]$$

Afterward, the mutual entropy *H_j_* obtained from Equations (12) and (13) using differential coefficient of mixing entropy for *p_j_*_+1_* [7,17]:

(14) $$H_{j}=I_{j}-I_{j+1}=\frac{\partial I_{j}}{\partial p_{j+1}*}\Delta p_{j+1}*\approx X\log\frac{p_{j+1}}{p_{j+1}*}\Delta p_{j+1}*=\log\frac{p_{j+1}}{p_{j+1}*}\Delta X_{j+1}*$$

Then, the chemical extracted average chemical work, <*w_j_*>, in (10) from (i) to (iv) in Section 3 was calculated using Equation (14) as follows:

(15) $$\langle w_{j}\rangle =\oint_{}k_{B}T\log\frac{p_{j+1}}{p_{j+1}*}dX_{j+1}*$$

Here the author defined *the expanded chemical potential* of the *j^th^* signal molecules in similar manner around the equilibrium state using *µ*^0^ at standard condition:

(16) $$\mu \left(X_{j+1}\right)=\mu^{0}\left(X_{j+1}\right)+k_{B}T\log p_{j+1}$$

(17) $$\mu \left(X_{j+1}*\right)=\mu^{0}\left(X_{j+1}*\right)+k_{B}T\log p_{j+1}*$$

Using the chemical potentials and the sufficient long signal duration of the *j^th^* step *τ_j_* (→ ∞), the author has:

(18) $$\frac{1}{\tau_{j}}\log\frac{p_{j+1}}{p_{j+1}*}=\frac{\mu \left(X_{j+1}\right)-\mu \left(X_{j+1}*\right)}{k_{B}T\tau_{j}}.$$

Previously, the author obtained the following result using the average entropy production rate (AEPR) *ζ_j_*, the current density of entropy production rate, *c_j_*_+1_ [7]:

(19) $$\zeta_{j}=\frac{c_{j+1}\Delta X_{j+1}*}{k_{B}T\tau_{j}}.$$

Here, *c_j_*_+1_ is given by chemical potential difference between species:

(20) $$c_{j+1}=\mu \left(X_{j+1}\right)-\mu \left(X_{j+1}*\right)$$

From Equations (15), (18) and (20), the author obtained the following:

(21) $$\langle w_{j}\rangle =\oint_{}c_{j+1}dX_{j+1}*=c_{j+1}\Delta X_{j+1}*$$

The integral symbol signifies the integration of the work through the cycle step. Likewise, the extracted work by the machinery between the *j^th^* and (*j* + 1)*^th^* reaction fields, *w*_−*j*_, in the opposite orientation in (ii) is given by:

(22) $$\langle w_{-j}\rangle =\oint_{}k_{B}T\log\frac{p_{j+1}*}{p_{j+1}}dX_{j+1}=\oint_{}k_{B}T\log\frac{p_{j+1}}{p_{j+1}*}dX_{j+1}*$$

(23) $$\langle w_{-j}\rangle =-c_{j+1}\Delta X_{j+1}=c_{j+1}\Delta X_{j+1}*$$

In this manner,

(24) $$\langle w_{j}\rangle =\langle w_{-j}\rangle ,$$

Then, from Equation (11),

(25) $$H_{j}=\frac{c_{j+1}\Delta X_{j+1}*}{k_{B}T}=\omega_{j+1}\Delta X_{j+1}*,$$

with

(26) $$\omega_{j+1}=\frac{c_{j+1}}{k_{B}T}$$

This *ω_j_*_+1_ represents the signal mobility [7]. Because the density of entropy signal current *c_j_*_+1_ is equivalent to the diffusion coefficient *D_j_*_+1_ according to our previous study [7], Equation (25) implies that the mutual entropy is carried by the signaling molecules:

(27) $$H_{j}=\frac{D_{j+1}\Delta X_{j+1}*}{k_{B}T}$$

Thus, the mutual entropy can be related to the diffusion process of signaling molecule.

## 5. Conclusions

Generally, cellular systems maintain homeostasis, in which fluctuations are minimized, while the extracellular stimuli induce the fluctuations in the system. Considering the feedback controller, signal transduction can be formulated according to the fluctuations in the concentration of signaling molecule, and the signal transduction level in the BSC may be evaluated quantitatively. In the *mechanicochemical* process, the difference between the chemical potentials of activated species and non-activated species is the source of the chemical work. The entire transfer step forms a cycle step. The entire average work <*w_j_*> done through the cycle by a given system is equal to the sum of the works by transferring the extensive amount Δ*X_j_*_+1_* through its conjugate potential as follows [18,19].

(28) $$\langle w_{j}\rangle =\oint_{C}\left[\mu \left(X_{j+1}\right)-\mu \left(X_{j+1}*\right)\right]dX_{j+1}*$$

Equations (15) and (22) can be considered as a form of Equation (28). This work formula in Equations (15) and (22) is consistent with the Inequality (1) [5,12,13]. The chemical work may be associated with AEPR during the cycle reaction of the steps. By measuring the kinetic parameter based on Formula (3), it is possible to actually compute the average work <*w_j_*> by entropy production in hydrolysis of ATP during the signal transduction. Using the probability of step *j* + 1, given step *j*, is defined as *p* (*j* + 1|*j*) and probability of step *j*, given step *j* + 1, is defined as *p* (*j* + 1|*j*), FT yields the following, using AEPR *ζ_j_*:

(29) $$\underset{\tau_{j}\to \infty }{\lim}\frac{1}{\tau_{j}}\log\frac{p\left(j+1|j\right)}{p\left(j|j+1\right)}=-\zeta_{j}$$

with

(30) $$\zeta_{j}≜\frac{1}{\tau_{j}}\int_{0}^{\tau_{j}}\Delta \zeta_{j}(s_{j})ds_{j}$$

Here, *s_j_* is an arbitrary parameter representing the progression of a signal event. The transitional rate of step *j* + 1, given step *j*, as *v* (*j* + 1|*j*) and the transitional rate of step *j*, given step *j* + 1, as *v* (*j*|*j* + 1) are defined. When the cell system stays at the detailed balance in the homeostasis, as follows:

(31) p(j+1|j)v(j+1|j)=p(j|j+1)v(j|j+1).

Therefore, from Equations (29) and (31):

(32) $$\underset{\tau_{j}\to \infty }{\lim}\frac{1}{\tau_{j}}\log\frac{v\left(j+1|j\right)}{v\left(j|j+1\right)}≃\zeta_{j}.$$

From Equations (19), (25) and (32), and the kinetic coefficient *k_j_* for *j^th^*-step in Formula (3), following is obtained:

(33) $$\underset{\tau_{j}\to \infty }{\lim}\frac{1}{\tau_{j}}\log\frac{k_{j}AX_{j}*X_{j+1}}{k_{-j}Ph_{j}X_{j+1}*}=\frac{c_{j+1}\Delta X_{j+1}*}{k_{B}T\tau_{j}}.$$

Then we have

(34) $$\log\frac{k_{j}AX_{j}*X_{j+1}}{k_{-j}Ph_{j}X_{j+1}*}≃H_{j}$$

Here, the author has the following from Equations (11) and (34):

(35) $$\langle w_{j}\rangle =k_{B}T\log\frac{k_{j}AX_{j}*X_{j+1}}{k_{-j}Ph_{j}X_{j+1}*}$$

By measuring the kinetic parameter based upon Equation (35), it is possible to actually compute the average work <*w*>. This simple relation may be verified by using experimental data as follows. There have been reports that chemical potential difference can be converted to work under isothermal status [20]. With this recent knowledge, this study attempted to present a model of the signal transduction step as a Szilard engine that can extract work. This modeling is actually effective for quantifying chemical work as described in the text. However, it is necessary to verify this modeling by further experimental studies.

In conclusion, the work that can be extracted in the signal-transduction step can be obtained using Inequality (1). Recently, many theories for computing protein–protein networks and gene expression networks have been developed [21,22]. Signaling entropy was recently investigated from the viewpoint of genome informatics [23,24], and its availability was confirmed [25,26]. In these studies, signaling entropy was defined using the transient probability obtained from each node in a network graph of the transcriptome profile of a single cell in order to quantify the gene activation levels of its molecular pathways. In the current study, the author aimed to connect these computational methods using informational thermodynamics and the kinetics of actual chemical reactions. The presented BSC model can describe the relationship between the chemical potential, mutual entropy, and work information, which is based on the information thermodynamics.
